# Psychometric Properties of the Metacognitions Questionnaire-30 (MCQ-30) in Older Norwegian Adolescents

**DOI:** 10.1007/s10578-025-01843-1

**Published:** 2025-04-23

**Authors:** Hanne Undheim Hoff, Odin Hjemdal, Silje Steinsbekk, Henrik Nordahl

**Affiliations:** 1 Department of Psychology, Norwegian University of Science and Technology, Trondheim, Norway; 2 St. Olavs Hospital, Division of Child and Adolescent Psychiatry, BUP Klostergata, Trondheim, Norway

**Keywords:** Anxiety, Adolescents, Depression, Measurement invariance, Metacognitive beliefs

## Abstract

Dysfunctional metacognitive beliefs have been established as a transdiagnostic correlate of anxiety and depression in adults and are considered a central mechanism of persistent emotional distress according to the metacognitive model of psychological disorders. However, the importance of metacognitions for distress and emotional disorder in adolescence is far less researched, and to investigate this further there is a need for reliable and valid assessment tools. The Metacognitions Questionnaire-30 (MCQ-30) might be a suitable and valid assessment tool in adolescents with the implication that it can be used to research the role of metacognitions in youth mental health and track how metacognitions change and relate to outcomes over time from adolescence to adulthood. We therefore aimed to examine the psychometric properties of the MCQ-30 in an Upper Secondary School-sample of 494 Norwegian adolescents, aged 16- to 18-years old. A confirmatory factor analysis indicated a good fit for the proposed five-factor structure and evidence for measurement invariance was supported across sex and groups of anxiety severity. The factors showed acceptable to good internal consistency and there was support for convergent validity. In conclusion, these findings indicate that the adult version of the MCQ-30 can be applied in Norwegian adolescents from 16-years old.

## Introduction

In the Self-regulatory executive function (S-REF) model [46,44], the mechanism proposed to underly psychopathology is a particular negative and rigid thinking style named the Cognitive Attentional Syndrome (CAS; [43]). The CAS includes top-down directed self-regulatory strategiesin the form of perseverative thinking patterns (e.g., worry/rumination), strategic attention towards threats (i.e., “threat monitoring”) and maladaptive coping behaviors (e.g., thought control, avoidance). The CAS is considered to result from dysfunctional beliefs about cognition (i.e., metacognitive beliefs), for example “worrying is uncontrollable” or “I need to control my thoughts” [43]. The clinical implication that naturally follows from the model is that psychological disorders are maintained by a common set of metacognitive beliefs and strategies, and that treatment that effectively modify these factors will be successful for a range of emotional disorders and comorbid symptoms [43].

To empirically test the role of dysfunctional metacognitions to emotional distress and disorders, valid and reliable assessment tools are needed. Since the publication of the S-REF model [46] several have been developed (see Capobianco and Nordahl [8] for an overview), but the most used and empirically supported is the metacognitions questionnaire (MCQ: [10]) and its 30-items shortened version (MCQ-30; [45]). The MCQ-30 assesses five subscales of metacognitive beliefs: (1) positive beliefs about worry; (2) negative beliefs about uncontrollability of thoughts and danger of worry; (3) cognitive confidence; (4) beliefs about the need to control thoughts; and (5) cognitive self-consciousness. The MCQ-30 has good psychometric properties in non-clinical (e.g., [19, 34]) and clinical (e.g., [13, 16]) adult populations. It has been important in establishing the role of dysfunctional metacognitive beliefs across a range of symptom domains and emotional disorders [7, 8, 15, 21, 38].

Future directions for research on the role of metacognitions in psychopathology include evaluating their role in the mental health of children and adolescents [8]. The prevalence of emotional disorders increases in adolescence [14], also at the symptoms level [26, 36]. Thus, there is a need to have psychometric sound and measurement invariant assessment tools of metacognitions that can be used in adolescents for research and clinical purposes. The application of the original MCQ-30 developed for adults in adolescence enables direct comparison of dysfunctional metacognitive beliefs with adult populations. Further, to be positioned to properly examine the role of metacognitions in the development of emotional disorder through adolescence, a strong psychometric measure of metacognitions is needed.

Myers et al. [27] conducted a systematic review of the MCQ and its derivates in children and adolescents and concluded that further psychometric research is needed to advance research on the role of metacognitions in youth mental health. More recently, Thingbak and colleagues [39] reviewed and synthesized the empirical research on the associations between metacognitive beliefs and anxiety and depression in youth. They identified 32 papers with a total of 9887 participants aged 7–18 years and concluded that metacognitive beliefs, positively correlate with anxiety and depression in youths and that clinical groups endorsed dysfunctional metacognitive beliefs more strongly compared to non-clinical groups (all except the domain for positive beliefs about worry). However, they identified a problem with moderate to high between-study heterogeneity in these results which they partly attributed to the use of different adaptations and versions of the MCQ which has not been appropriately anchored or psychometrically evaluated. Hence, the need for psychometric evaluation of measures assessing metacognitions is still warranted to secure that the same concepts are being investigated and to reduce error of measurement.

Declarative metacognitive knowledge is considered well-developed in older adolescents, [33]. Applying the original adult-version of MCQ-30 in this age group is therefore most likely suitable. Further, using the original MCQ-30 without modifications has the advantage of allowing us to compare results across late adolescence and adulthood, and help overcome some of the issues associated with MCQ derivates [27]. To the best of our knowledge, only one study has evaluated the psychometric properties of the MCQ-30 in adolescents. Li et al. [23] included 2827 adolescents (aged 11 to 18 years) in China and found support for the proposed five-factor structure of the MCQ-30. The factors were found to be internally consistent, positively correlated with symptoms of anxiety and depression, and measurement invariant on three levels (configural, metric and scalar) across several demographic variables and high/low levels of anxiety/depression. Because there are differences between Eastern and Western cultures (e.g., collectivistic vs. individualistic), which may also affect adolescent development and functioning, it is important to test whether the MCQ-30 instrument is valid across such differing societies.

With an aim to facilitate research on the role of dysfunctional metacognitions in adolescents, we set out to evaluate the psychometric properties of the MCQ-30 in older Norwegian adolescents aged 16 to 18 years. We hypothesize that the original five-factor solution will provide a good fit to the data and that measurement invariance (across sex and anxiety levels) will be displayed. We further expect the MCQ-30 subscales to show adequate internal consistency and to find support for convergent validity.

## Methods

### Procedure and Participants

Five Upper Secondary Schools in Middle-Norway were contacted and informed about the study. Four agreed to participate, whereas the fifth school declined due to time restraints. Dates for data-collection were arranged with teachers. The sample was therefore gathered at convenience. Adolescents aged 16 to 18 years old were invited to participate in a survey to evaluate the MCQ-30 and its associations with anxiety and depression. Participants received written and oral information about the study and signed a written consent form. They were also informed that they were eligible for winning one out of three “Sony around-ear Headphones”, by taking part in the study. Proficiency in Norwegian was a criterion for inclusion, met by all pupils enquired for participation. The participants completed the questionnaires with pen and paper, for a duration of 20–30 min, during school hours. Project staff members or teachers were present during completion, to assist the participants if needed (e.g., how to correctly sign the consent form). Four-hundred and ninety-four individuals participated of which 276 were females (55,9%) and 218 males (44,1%). Two-hundred and sixty (52,6%) were 16-years old, 108 (21.9%) were 17-years old, and 126 (25,5%) were 18-years old. The study was approved by the Regional Committees for Medical and Health Research Ethics (ref.nr. 606317, REK-Midt).

### Measures

The Metacognitions questionnaire-30 (MCQ-30; [45]) assesses beliefs about thoughts and cognition and consist of 30 items rated on a four-point scale from 1 (“I do not agree”) to 4 (“I strongly agree”). There are five subscales consisting of six items each: (1) Positive beliefs about worry (e.g. “worrying helps me to solve problems”), (2) Negative beliefs about the uncontrollability of thoughts and danger of worry (e.g. “when I start to worry I cannot stop”), (3) Cognitive confidence (e.g. “I do not trust my memory”), (4) Need to control thoughts (e.g. “I should be in control of my thoughts all of the time”), (5) Cognitive self-consciousness (e.g. “I am constantly aware of my thinking”). MCQ-30 has been broadly validated for use in adults (e.g. [19, 34, 45]). The MCQ-30 was developed in English, but the Norwegian translation of MCQ-30 has been validated in adults [16], 29] and is identical to the version used in the present study.

The Screen for Child Anxiety related Emotional Disorders (SCARED; [5, 6] measures aolescents and is frequently used in child and adolescent psychiatric outpatient clinics in Norway and world-wide. The psychometric properties of SCARED have been found to be robust in both clinical and community samples (e.g., [12, 37, 41, 42]). The applied version of SCARED [5] has 41 items that are rated as either 0 (“almost never”), 1 (“sometimes”) or 2 (“often”). In testing convergent validity in this study, the SCARED total score was applied as a continuous variable (*α* = 0.96, ω = 0.96).

Short Mood and Feelings Questionnaire (SMFQ; [1]) is a frequently used questionnaire to measure depressive symptoms in children and adolescents aged 8–18 years old. The SMFQ is the short version of the original Mood and feelings Questionnaire (MFQ; [1]), recommended by the National Institute for Health and Clinical Excellence (NICE guidelines [28]) for the screening of depression in children and adolescents. The short version consists of 13 items rated on a 3-point scale from 0 “not correct” to 2 “correct”. The SMFQ is found to have a very strong correlation with the full 33-item MFQ and is considered an effective measurement for assessing adolescents in primary care or research settings, limiting survey-time (α = 0.90, ω = 0.90).

## Statistical Analyses

Statistical analyses were conducted using JASP 0.18.3. To evaluate the proposed five-factor structure of the MCQ-30, a confirmatory factor analysis (CFA) was performed. We used robust estimation and the diagonal weighted least squares (DWLS) estimator recommended for ordinal data (Li [22]), combined with listwise deletion for missing data. In JASP, listwise deletion is compatible with selecting a DWLS estimator, and considered appropriate as the missingness at the item level in our data was below 0.4%.

According to recommendations for assessing model fit [18], we included the following fit indices: the Comparative Fit Index (CFI; [3]), the Tucker-Lewis Index (TLI; [40]), the Root Mean Square Error of Approximation (RMSEA; [35]) and the Standardized Root Mean Square Residual (SRMR; [4, 20]). Recommended cut-off values for CFI and TLI are 0.90 for acceptable model fit and 0.95, indicating very good model fit. Furthermore, recommended cut-off values for RMSEA are below or close to 0.06, and SRMR should be less than 0.08 [18].

Secondly, measurement invariance was tested on four different levels for both groups (configural, metric, scalar and strict). Measurement invariance can indicate whether the same construct is being measured across some specified groups or over time which is particularly important in developmental research [24]. The MCQ-30 structure was tested for measurement invariance across sex (male and female) and severity of anxiety symptoms (two groups consisting of those below versus at or above the cut-off score of 25 on SCARED). Configural invariance measure is an indication of whether the overall factor structure is the same between groups; metric invariance is a test of the item factor loadings between groups; and the scalar invariance indicates the similarity of item thresholds between investigated groups. Strict invariance indicates that loadings, measurement intercepts and measure residual variances are equal across groups [25]). When testing for measurement invariance, examining the change in Comparative Fit Index (CFI; [3]) and the Root mean square error of approximation (RMSEA; [35]) between models (ΔCFI, ΔRMSEA) are recommended as suitable measures (Chen [11]). Criteria for assessing invariance can vary depending on sample size or unequal sample size between groups, but a ΔCFI value less than 0.01 and a ΔRMSEA less than 0.015 are commonly applied criteria of non-invariance [23, 32].

Thirdly, means with standard deviations on the MCQ-30 total score and the five subscales, and their internal consistencies (Cronbach`s alpha and McDonald’s Omega Coefficient) were calculated.

Finally, convergent validity was tested by examining bivariate correlations between MCQ-30 subscales, anxiety (SCARED) and depression (SMFQ). Positive relationships between the five factors and symptoms of anxiety and depression were used as indicators of convergent validity.

## Results

### Confirmatory Factors Analysis

**Fig. 1:**
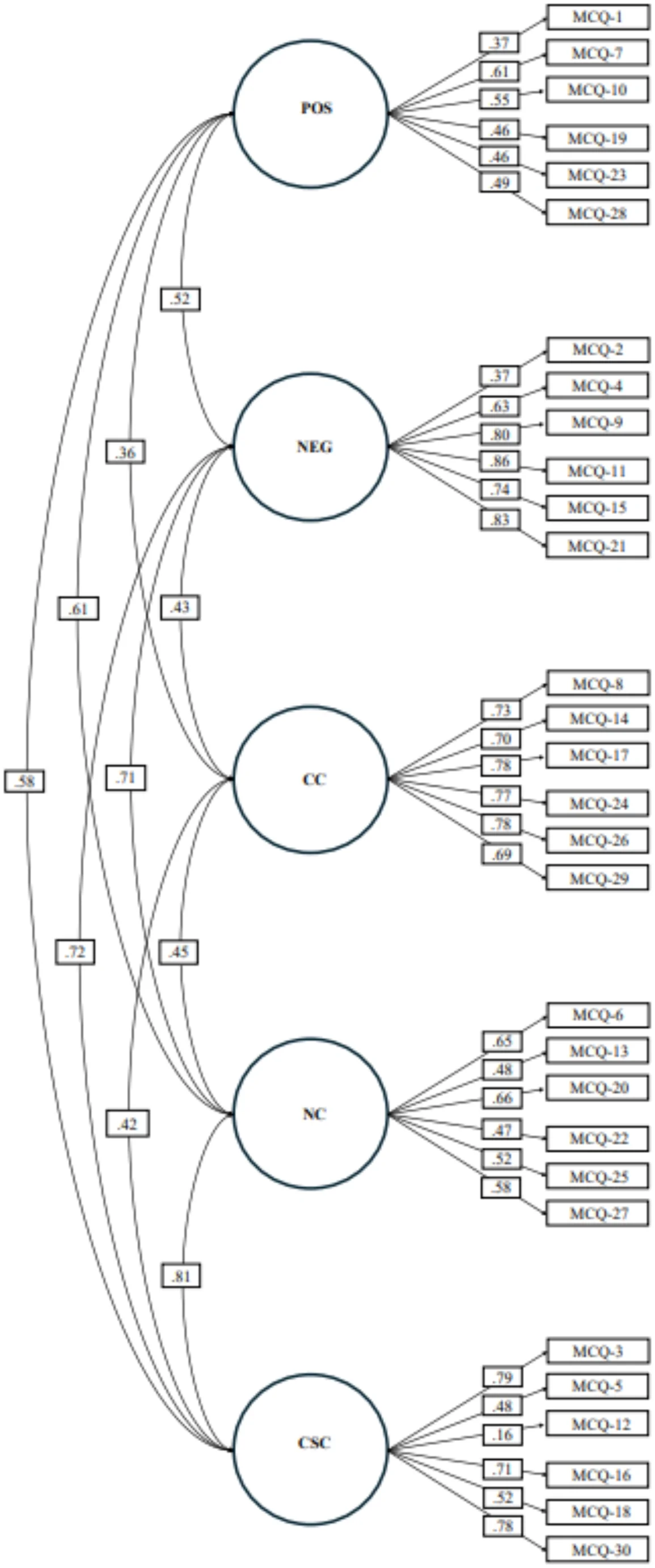
Confirmatory factor analysis (*N* = 494). POS = Positive beliefs about worry; NEG = Negative beliefs about uncontrollability of thoughts and danger of worry, CC = Cognitive confidence; NC = Need for control; CSC = Cognitive self-consciousness

The CFA indicated that the five-factor model has a very good fit to the data (χ^2^ (395) = 572.07, *p* <.001; CFI = 0.987, TLI = 0.986, RMSEA [90% CI] = 0.031 [0.026 − 0.037], SRMR = 0.056). As displayed in Fig. 1, all the separate items of the MCQ-30 were influenced by their belonging factors. Furthermore, all the factors were significantly correlated (*p* <.01) and the factor loadings for each of the five factors were greater than 0.35, with the exception of one item (item MCQ12 loading on the CSC factor) (see Fig. 1).

## Measurement Invariance

Results from measurement invariance testing of the five-factor model across two groups of sex (male and female) and severity of anxiety (below versus above SCARED cut-off) are presented in Table 1.

**Table 1:** Measurement invariance of the five-factor model (*N* = 494)

Model	CFI	TLI	RMSEA (90% CI)	Δ CFI	Δ RMSEA
**Sex**					
Configural	0.997	0.997	0.014 (0.000–0.025)		
Metric	0.994	0.994	0.020 (0.006–0.029)	0.003	−0.006
Scalar	0.991	0.991	0.025 (0.015–0.032)	0.003	−0.005
Strict	0.989	0.989	0.028 (0.020–0.035)	0.002	−0.003
**Anxiety**					
Configural	0.976	0.974	0.034 (0.027–0.040)		
Metric	0.975	0.974	0.034 (0.027–0.040)	0.001	0.000
Scalar	0.966	0.965	0.039 (0.033–0.045)	0.009	−0.005
Strict	0.957	0.957	0.044 (0.038–0.049)	0.009	−0.005

CFI = Comparative Fit Index; TLI = Tucker-Lewis Index; RMSEA = Root Mean Square error of approximation; CI = Confidence Interval; Δ = change

The testing of configural, metric, scalar and strict measurement invariance showed that the factor structure, the factor loadings and the indicator thresholds of the MCQ-30 were the same, across sex and anxiety level.

### Descriptive Data and Internal Consistency

In the present sample, the mean total score for MCQ-30 was 53.31, with a standard deviation (*SD*) of 15.23. The mean and SDs for each of the subscales were as follows: Positive beliefs about worry (POS); 9.29 (*SD* 3.23), Negative beliefs about uncontrollability of thoughts and danger of worry (NEG); 10.76 (*SD* 4.62), Cognitive confidence (CC); 10.86 (*SD* 4.79), Need to control thoughts (NC); 10.78 (*SD* 3.93) and Cognitive self-consciousness (CSC); 12.01 (*SD* 4.00). The internal consistency for the MCQ-30 total score was found to be very good (*α* = 0.93, ω = 0.93) and ranged from acceptable to good for the subscales (POS *α* = 0.84, ω = 0.84, NEG *α* = 0.89, ω = 0.90 CC *α* = 0.90, ω = 0.90, NC *α* = 0.77, ω = 0.78 CSC *α* = 0.78, ω = 0.80).

## Convergent Validity

To test convergent validity, each of the five MCQ-30 subscales were correlated with anxiety (SCARED) and depression (SMFQ) (presented in Table 2). All five subscales of the MCQ-30 were significantly and positively correlated with anxiety and depression, showing moderate to strong associations. Negative beliefs about uncontrollability of thoughts and danger of worry had the strongest relationship to anxiety and depression symptoms among the metacognitive belief factors.

**Table 2:** Bivariate correlations between the MCQ-30 subscales, anxiety- and depression symptoms (*N* = 494)

	2	3	4	5	6	7
1. POS	0.442*	0.293*	0.496*	0.489*	0.401*	0.345*
2. NEG		0.407*	0.616*	0.579*	0.761*	0.667*
3. CC			0.378*	0.318*	0.452*	0.446*
4. NC				0.647*	0.523*	0.526*
5. CSC					0.499*	0.456*
6. Anxiety						0.726*
7. Depression						

*p <.001; POS = Positive beliefs; NEG = Negative beliefs about uncontrollability of thoughts and danger of worry, CC = Cognitive confidence; NC = Need for control; CSC = Cognitive self-consciousness

## Discussion

The aim of the present study was to evaluate the psychometric properties of the MCQ-30 in a sample of Norwegian adolescents. As hypothesized, the fit indices indicated that the five-factor model had an excellent fit, with the exception of a significant chi-square. A significant chi-square indicates a poor fitting model, but this statistic is very sensitive to sample sizes and models with larger numbers of observed variables [17]. Four levels of measurement invariance were tested and in sum the MCQ-30 five-factor structure were found to have measurement equivalence across sex (male/female) and across two levels of anxiety. The factors demonstrated acceptable to good reliability, and significant and positive relationships with anxiety and depressive symptoms indicating convergent validity.

The current results are in line with a study of Chinese adolescents [23], as well as previous findings from Norwegian adults [16, 29]. Finding good psychometric properties of the MCQ-30 in a Scandinavian/Western adolescent sample indicates that the measure is relatively culturally independent, although future studies from other parts of the world are needed to substantiate such claim.

The original MCQ-30 with its proposed five-factor structure holds in a group of 16- to 18-year-olds, which suggest that the measure can be used in studies of dysfunctional metacognitions in this age-group and is applicable in clinical practice. The findings justify the use of MCQ-30 in observational and clinical studies. This is important because longitudinal studies in normally developing adolescents are needed to gain insight into the development of metacognitive beliefs across adolescence, and we need intervention studies examining the effect of metacognitive therapy and other methods that may affect metacognitions. This type of research relies on access to validated measurement tools, such as the MCQ-30.

In the present sample, one of the items (MCQ12; “I monitor my thoughts”) showed a low loading to its suggested factor (CSC). The same finding has been reported in two previous studies evaluating the MCQ-30 using network analysis in Norwegian adults [2, 29]) The aim of the current study was to test if the established factor structure was confirmed in the present sample, which it did. If this was not the case, it would have been appropriate to go with exploratory approaches and potentially modifying the measure. However, a modification would not allow for comparing findings across studies, which would represent a serious limitation. While metacognitive beliefs assessed with the MCQ-30 theoretically can be distinguished in subcategories, it is expected that factors and its items will be interconnected. Using a network approach to evaluate the structure of the MCQ-30 and not relying on a common factor model, [29] found that the MCQ12 item shared positive edges with both the CSC and the NC cluster and argue “I monitor my thoughts” are conceptually linked to more general beliefs about the importance and power of thoughts as assessed with NC. One option for further research on the MCQ-30 in adolescents is to explore its structure and centrality of specific metacognitions taking interdependence and mutual influence between the items and factors into account.

Measurement invariance (MI) tests assess the psychometric equivalence of a construct across groups and support for measurement invariance is a prerequisite to evaluate for example group differences or to assess the relations between theoretical concepts over time (e.g., [31]. We tested four levels of MI (i.e., configural, metric, scalar, strict) and found support for measurement invariance across sex (i.e., male and female) and anxiety level (below versus above clinical cut-off on the SCARED). This finding indicates that the MCQ-30 assess the same constructs in females and males, and among those below and above a cut-off for clinical anxiety which means that we can apply the measure to assess gender differences and compare individuals with different levels of anxiety (i.e., the five MCQ subscales does not depend on anxiety level). This result is in line with the study by [23] who also reported that the adult version of the MCQ-30 demonstrated measurement invariance (configural, metric, scalar) in adolescents. Adding to the [23] study, we also tested and found support for strict invariance, meaning that the sum of specific variance and error variance is similar across the tested groups.

Consistent with findings from a Chinese study [23], the internal consistency for the MCQ-30 total scale and the five subscales ranged from acceptable to very good in this sample. The previous review of the psychometric properties of MCQ derivates in youth reported mixed results for internal reliability, especially for the NC subscale [27]. While the inconsistent findings could result from small samples sizes, as pointed out by Li et al. [23], another explanation is that the MCQ derivates are not well enough adjusted. Our results indicate that applying the original MCQ-30 in older adolescents is one way to overcome this issue and that it provides a reliable measurement of metacognitive factors.

Our results support convergent validity of the MCQ-30 factors as they all showed positive and significant correlations with anxiety and depression. In line with metacognitive theory (Wells and Matthews [46]) and the recent review of metacognitions and their associations with anxiety and depression in youth [39], the factor negative beliefs about uncontrollability of thoughts and danger of worry (NEG) showed the strongest correlation with symptoms. This observation aligns with the suggestion that negative metacognitive beliefs are the strongest correlate of emotional distress symptoms due to its close relationship with the CAS. In turn the CAS is considered the more proximal influence on emotional distress symptoms compared to metacognitive beliefs [43].

There are several limitations associated with our study that should be acknowledged. We relied on a convenience sample, which limits the possibility to generalize to other samples. Support of measurement invariance is restricted to two grouping variables, and analysis using depression as a grouping variable was not possible due to a low number of individuals reporting elevated depression. The findings are limited to older adolescents, and further investigation on younger adolescents will be useful in exploring validity of the measure for an even wider application. Furthermore, there is a need to evaluate the psychometrics of the MCQ-30 in clinical samples. Moreover, we used only one assessment point, and there is a need to assess the test-retest reliability of the factors, to evaluate whether they are responsive to therapeutic interventions, and to test measurement invariance over time.

In conclusion, the original MCQ-30 can be applied to Norwegian adolescents aged 16–18 years old to assess dysfunctional metacognitive beliefs. This finding enables further research on the role for dysfunctional metacognitions which in the long run has the potential to advance formulation and interventions to address psychological vulnerability and disorders in adolescents.

## Data Availability

The data is available upon reasonable request to the corresponding author.

## References

[1] Angold A, Costello EJ, Messer SC, Pickles A, Winder F, Silver D (1995) Development of a short questionnaire for use in epidemiological studies of depression in children and adolescents. Int J Methods Psychiatr Res 5(4):237–249

[2] Anyan F, Nordahl H, Hjemdal O (2023) The network structure of dysfunctional metacognitions, CAS strategies, and symptoms. Cogent Psychol 10(1). 10.1080/23311908.2023.2205258

[3] Bentler PM (1990) Comparative fit indexes in structural models. Psychol Bull 107(2):238–246. 10.1037/0033-2909.107.2.238 2320703

[4] Bentler PM (1995) Eq. 5 [Computer Program]. Multivariate Software Inc, Encino, CA

[5] Birmaher B, Khetarpal S, Brent D, Cully M, Balach L, Kaufman J, Neer SM (1997) The screen for child anxiety related emotional disorders (SCARED): scale construction and psychometric characteristics. J Am Acad Child Adolesc Psychiatry 36(4):545–553. 10.1097/00004583-199704000-00018 9100430

[6] Birmaher B, Brent DA, Chiappetta L, Bridge J, Monga S, Baugher M (1999) Psychometric properties of the screen for child anxiety related emotional disorders (SCARED): A replication study. J Am Acad Child Adolesc Psychiatry 38(10):1230–1236. 10.1097/00004583-199910000-00011 10517055

[7] Cano-López JB, García-Sancho E, Fernández-Castilla B, Salguero JM (2021) Empirical evidence of the metacognitive model of rumination and depression in clinical and nonclinical samples: A systematic review and meta-analysis. Cogn Therapy Res 46367–392. 10.1007/s10608-021-10260-2

[8] Capobianco L, Nordahl H (2023) A brief history of metacognitive therapy: from cognitive science to clinical practice. Cogn Behav Pract 30(1):45–54. 10.1016/j.cbpra.2021.11.002

[9] Capobianco L, Faija C, Husain Z, Wells A (2020) Metacognitive beliefs and their relationship with anxiety and depression in physical illnesses: A systematic review. PLoS ONE 15(9):e0238457. 10.1371/journal.pone.0238457 PMC7500039 32911486

[10] Cartwright-Hatton S, Wells A (1997) Beliefs about worry and intrusions: the Meta-Cognitions questionnaire and its correlates. J Anxiety Disord 11(3):279–296. 10.1016/S0887-6185(97)00011-X 9220301

[11] Chen FF (2007) Sensitivity of goodness of fit indexes to lack of measurement invariance. Struct Equation Modeling: Multidisciplinary J 14(3):464–504. 10.1080/10705510701301834

[12] Essau CA, Muris P, Ederer EM (2002) Reliability and validity of the spence children’s anxiety scale and the screen for child anxiety related emotional disorders in German children. J Behav Ther Exp Psychiatry 33(1):1–18. 10.1016/S0005-7916(02)00005-8 12389796

[13] Faija CL, Reeves D, Heal C, Wells A (2020) Metacognition in cardiac patients with anxiety and depression: psychometric performance of the metacognitions questionnaire 30 (MCQ-30). Front Psychol 11:1064. 10.3389/fpsyg.2020.01064 PMC7264260 32528387

[14] Ghandour RM, Sherman LJ, Vladutiu MMA, Lynch SE, Bitsko RH, Blumberg SJ (2019) Prevalence and treatment of depression, anxiety, and conduct problems in US children. J Pediatr 206:256–267. 10.1016/j.jpeds.2018.09.021 PMC6673640 30322701

[15] Gkika S, Wittkowski A, Wells A (2018) Social cognition and metacognition in social anxiety: A systematic review. Clin Psychol Psychother 25(1):10–30. 10.1002/cpp.2127 28836318

[16] Grötte T, Solem S, Myers SG, Hjemdal O, Vogel PA, Güzey IC, Hansen B, Nordahl HM, Fisher P (2016) Metacognitions in obsessive-compulsive disorder: A psychometric study of the metacognitions Questionnaire-30. J Obsessive-Compulsive Relat Disorders 11:82–90. 10.1016/j.jocrd.2016.09.002

[17] Hair JF, Black WC, Babin BJ, Anderson RE (2014) Multivariate data analysis. Harlow, Essex, Seventh ed. Pearson new international

[18] Hu L-T, Bentler PM (1999) Cutoff criteria for fit indexes in covariance structure analysis: conventional criteria versus new alternatives. Struct Equation Modeling: Multidisciplinary J 6(1):1–55. 10.1080/10705519909540118

[19] Huntley CD, Young B, Smith CT, Jha V, Fisher P (2020) Assessing metacognitive beliefs in test anxiety: psychometric properties of the metacognitions questionnaire, 30 (MCQ-30) among university students. Curr Psychol 41:1425–1433. 10.1007/s12144-020-00662-y

[20] Jöreskog KG, Sörbom D (1988) LISREL 7. A guide to the program and applications, 2nd edn. International Education Services

[21] Keen E, Kangas M, Gilchrist GT (2022) A systematic review evaluating metacognitive beliefs in health anxiety and somatic distress. Br J Health Psychol 27(4):1398–1422. 10.1111/bjhp.12609 PMC9796692 35746856

[22] Li C-H (2016) Confirmatory factor analysis with ordinal data: comparing robust maximum likelihood and diagonally weighted least squares. Behav Res Methods 48(3):936–949. 10.3758/s13428-015-0619-7 26174714

[23] Li F, Yuan D, Gao C, Xiong K, Geng F, Zhang L (2023) Validity and reliability of the metacognitions Questionnaire-30 (MCQ-30) among Chinese adolescents. Child Psychiatry Hum Dev. 10.1007/s10578-023-01625-7 37917240

[24] Liu Y, Millsap RE, West SG, Tein J-Y, Tanaka R, Grimm KJ (2016) Testing measurement invariance in longitudinal data with ordered-categorical measures. American Psychological Association 10.1037/met0000075 PMC5121102 27213981

[25] Millsap RE (2011) Statistical approaches to measurement invariance. New York: Routledge Res Methods 15(2):167–198

[26] Morken IS, Viddal KR, Ranum B, Wichstrøm L (2021) Depression from preschool to adolescence–five faces of stability. J Child Psychol Psychiatry 62(8):1000–1009. 10.1111/jcpp.13362 33242346

[27] Myers SG, Solem S, Wells A (2019) The metacognitions questionnaire and its derivatives in children and adolescents: A systematic review of psychometric properties. Front Psychol 10:1871. 10.3389/fpsyg.2019.01871 PMC6737041 31551843

[28] NICE Guidelines (2005) 1.1.13. National Institute for Health and Care Excellence (NICE) guideline number NG134. https://www.nice.org.uk/guidance/NG134

[29] Nordahl H, Hjemdal O, Hagen R, Nordahl HM, Wells A (2019) What Lies beneath trait-anxiety? Testing the self-regulatory executive function model of vulnerability. Front Psychol 10:122. 10.3389/fpsyg.2019.00122 PMC6371045 30804834

[30] Nordahl H, Anyan F, Hjemdal O, Wells A (2022) The network structure of dysfunctional metacognition: analysis of the MCQ-30. Acta Psychol 227:103622. 10.1016/j.actpsy.2022.103622 35643016

[31] Putnick DL, Bornstein MH (2016) Measurement invariance conventions and reporting: the state of the Art and future directions for psychological research. Dev Rev 41:71–90. 10.1016/j.dr.2016.06.004 PMC5145197 27942093

[32] Rutkowski L, Svetina D (2014) Assessing the hypothesis of measurement invariance in the context of large-scale international surveys. Educ Psychol Meas 74(1):31–57. 10.1177/0013164413498257

[33] Schneider W (2008) The development of metacognitive knowledge in children and adolescents: major trends and implications for education. Mind Brain Educ 2(3):114–121. 10.1111/j.1751-228X.2008.00041.x

[34] Spada MM, Mohiyeddini C, Wells A (2008) Measuring metacognitions associated with emotional distress: factor structure and predictive validity of the metacognitions questionnaire 30. Pers Indiv Differ 45:238–242. 10.1016/j.paid.2008.04.005

[35] Steiger JH, Lind JC (1980) Statistically based tests for the number of common factors. *Handout for a presentation delivered at the annual meeting of the Psychometric Society*. May 1980, Iowa City, IA

[36] Steinsbekk S, Ranum B, Wichstrøm L (2022) Prevalence and course of anxiety disorders and symptoms from preschool to adolescence: a 6-wave community study. J Child Psychol Psychiatry 63(5):527–534. 10.1111/jcpp.13487 34318492

[37] Su L, Wang K, Fan F, Su Y, Gao X (2008) Reliability and validity of the screen for child anxiety related emotional disorders (SCARED) in Chinese children. J Anxiety Disord 22(4):612–621. 10.1016/j.janxdis.2007.05.011 17628391

[38] Sun X, Zhu C, So SHW (2017) Dysfunctional metacognition across psychopathologies: a meta-analytic review. Eur Psychiatry 45:139–153. 10.1016/j.eurpsy.2017.05.029 28763680

[39] Thingbak A, Capobianco L, Wells A, Skytte O, Toole M (2024) Relationships between metacognitive beliefs and anxiety and depression in children and adolescents: A meta-analysis. J Affect Disord 361:36–50. 10.1016/j.jad.2024.05.123 38815761

[40] Tucker LR, Lewis C (1973) A reliability coefficient for maximum likelihood factor analysis. Psychometrika 38:1–10. 10.1007/BF02291170

[41] Vigil-Colet A, Canals J, Cosí S, Lorenzo-Seva U, Ferrando PJ, Hernández-Martínez C, Jané C, Viñas F, Doménech E (2009) The factorial structure of the 41-item version of the screen for child anxiety related emotional disorders (SCARED) in a Spanish population of 8 to 12 years-old. Int J Clin Health Psychol 9(2):313–327

[42] Weitkamp K, Romer G, Rosenthal S et al (2010) German screen for child anxiety related emotional disorders (SCARED): reliability, validity, and cross-informant agreement in a clinical sample. Child Adolesc Psychiatry Mental Health 4:19. 10.1186/1753-2000-4-19 PMC2912250 20591137

[43] Wells A (2009) Metacognitive therapy for anxiety and depression. Guilford Press, New York, NY

[44] Wells A (2019) Breaking the cybernetic code: Understanding and treating the human metacognitive control system to enhance mental health. Front Psychol 10:2621. 10.3389/fpsyg.2019.02621 PMC6920120 31920769

[45] Wells A, Cartwright-Hatton S (2004) A short form of the metacognitions questionnaire: properties of the MCQ-30. Behav Res Ther 42(4):385–396. 10.1016/s0005-7967(03)00147-5 14998733

[46] Wells A, Matthews G (1994) Attention and emotion: A clinical perspective. Lawrence Erlbaum Associates, Inc

